# Epigallocatechin gallate (EGCG) reduces the intensity of pancreatic amyloid fibrils in human islet amyloid polypeptide (hIAPP) transgenic mice

**DOI:** 10.1038/s41598-017-18807-8

**Published:** 2018-01-18

**Authors:** Andras Franko, Diana C. Rodriguez Camargo, Annett Böddrich, Divita Garg, Andres Rodriguez Camargo, Birgit Rathkolb, Dirk Janik, Michaela Aichler, Annette Feuchtinger, Frauke Neff, Helmut Fuchs, Erich E. Wanker, Bernd Reif, Hans-Ulrich Häring, Andreas Peter, Martin Hrabě de Angelis

**Affiliations:** 1 Institute of Experimental Genetics, Helmholtz Zentrum München, Neuherberg, Germany; 2 Department of Internal Medicine IV, Division of Endocrinology, Diabetology, Angiology, Nephrology and Clinical Chemistry, University Hospital Tübingen, Tübingen, Germany; 3 German Center for Diabetes Research (DZD e.V.), Neuherberg, Germany; 4 Institute for Diabetes Research and Metabolic Diseases of the Helmholtz Centre Munich at the University of Tübingen, Tübingen, Germany; 5 Helmholtz Zentrum München, Neuherberg, Germany; 6 Munich Center for Integrated Protein Science (CIPS-M) at Department Chemie, Technische Universität München (TUM), Freising, Germany; 7 Max-Delbrück-Centrum für Molekulare Medizin, Berlin, Germany; 8 Department of Molecular and Experimental Medicine, The Scripps Research Institute, La Jolla, CA 92037 USA; 9 Institute of Pathology, Helmholtz Zentrum München, Neuherberg, Germany; 10 Research Unit Analytical Pathology, Helmholtz Zentrum München, Neuherberg, Germany; 11 Center of Life and Food Sciences Weihenstephan, Technische Universität München, Freising, Germany; 12 Institute of Molecular Animals Breeding and Biotechnology, Ludwig-Maximilians-Universität München, Munich, Germany

## Abstract

The formation of amyloid fibrils by human islet amyloid polypeptide protein (hIAPP) has been implicated in pancreas dysfunction and diabetes. However, efficient treatment options to reduce amyloid fibrils *in vivo* are still lacking. Therefore, we tested the effect of epigallocatechin gallate (EGCG) on fibril formation *in vitro* and *in vivo*. To determine the binding of hIAPP and EGCG, *in vitro* interaction studies were performed. To inhibit amyloid plaque formation *in vivo*, homozygous (tg/tg), hemizygous (wt/tg), and control mice (wt/wt) were treated with EGCG. EGCG bound to hIAPP *in vitro* and induced formation of amorphous aggregates instead of amyloid fibrils. Amyloid fibrils were detected in the pancreatic islets of tg/tg mice, which was associated with disrupted islet structure and diabetes. Although pancreatic amyloid fibrils could be detected in wt/tg mice, these animals were non-diabetic. EGCG application decreased amyloid fibril intensity in wt/tg mice, however it was ineffective in tg/tg animals. Our data indicate that EGCG inhibits amyloid fibril formation *in vitro* and reduces fibril intensity in non-diabetic wt/tg mice. These results demonstrate a possible *in vivo* effectiveness of EGCG on amyloid formation and suggest an early therapeutical application.

## Introduction

Correct protein folding is essential for normal cellular function and many diseases are associated with protein misfolding^1^. Misfolded or misassembled proteins form cross-β-sheet fibrils, the so called amyloid deposits^2^. Human islet amyloid polypeptide (hIAPP) is highly amyloidogenic, and hIAPP fibrils are found *post-mortem* in 40–90% of patients with type 2 diabetes (T2D)^3^. hIAPP is produced in pancreatic beta-cells and it is co-secreted with insulin^2^. hIAPP inhibits insulin secretion and plays a possible role in gastric emptying^4,5^. Many studies suggest that hIAPP fibril formation contributes to the pathophysiology of T2D by inducing beta-cell dysfunction and apoptosis^3,6,7^. Due to the current hypothesis the initial amyloid fibril formation is intracellular, which is followed by the rupture of cellular membrane and due to further seeding processes, extracellular amyloid deposits are accumulated^4,8^. Currently the treatment options of amyloid diseases are limited and effective drugs inhibiting hIAPP amyloid formation *in vivo* are scarce^5^.

The polyphenol epigallocatechin gallate (EGCG) is one of the major active components of green tea. EGCG treatment has been demonstrated to be beneficial inhibiting calcitonin, amyloid-β and α-synuclein amyloid formation^9–11^ and it was also able to inhibit hIAPP amyloidogenesis *in vitro*^12–14^. Although charged side chains have been shown to participate in EGCG-protein interaction^15^, the exact binding site for EGCG in the hIAPP polypeptide as well as the structure of the hIAPP-EGCG complex has not been identified experimentally yet^1,5^. Furthermore, green tea catechins and EGCG were shown to ameliorate diabetes and to improve metabolic phenotype in rodent models of diabetes^16,17^. Human studies reported that green tea has beneficial effects on metabolic syndrome^18^ and could improve blood glucose and hemoglobin A1c levels in patients with T2D in some^19,20^ but not in all studies^21^. To understand the mechanism of pancreatic fibril formation and its inhibition with small inhibitor molecules, we have investigated hIAPP amyloidogenesis in the presence of EGCG. Our aim was i.) to identify the binding site between EGCG and hIAPP using NMR studies, ii.) to resolve the molecular structure of EGCG-bound hIAPP and iii.) to study the effectivity of EGCG on hIAPP amyloid fibrils *in vivo* by applying EGCG on hIAPP transgenic mice, which is a well described mouse model characterized by hIAPP overexpression and diabetes^22^.

## Results

### hIAPP transgenic mice, a mouse model to study amyloid formation

**Figure 1:**
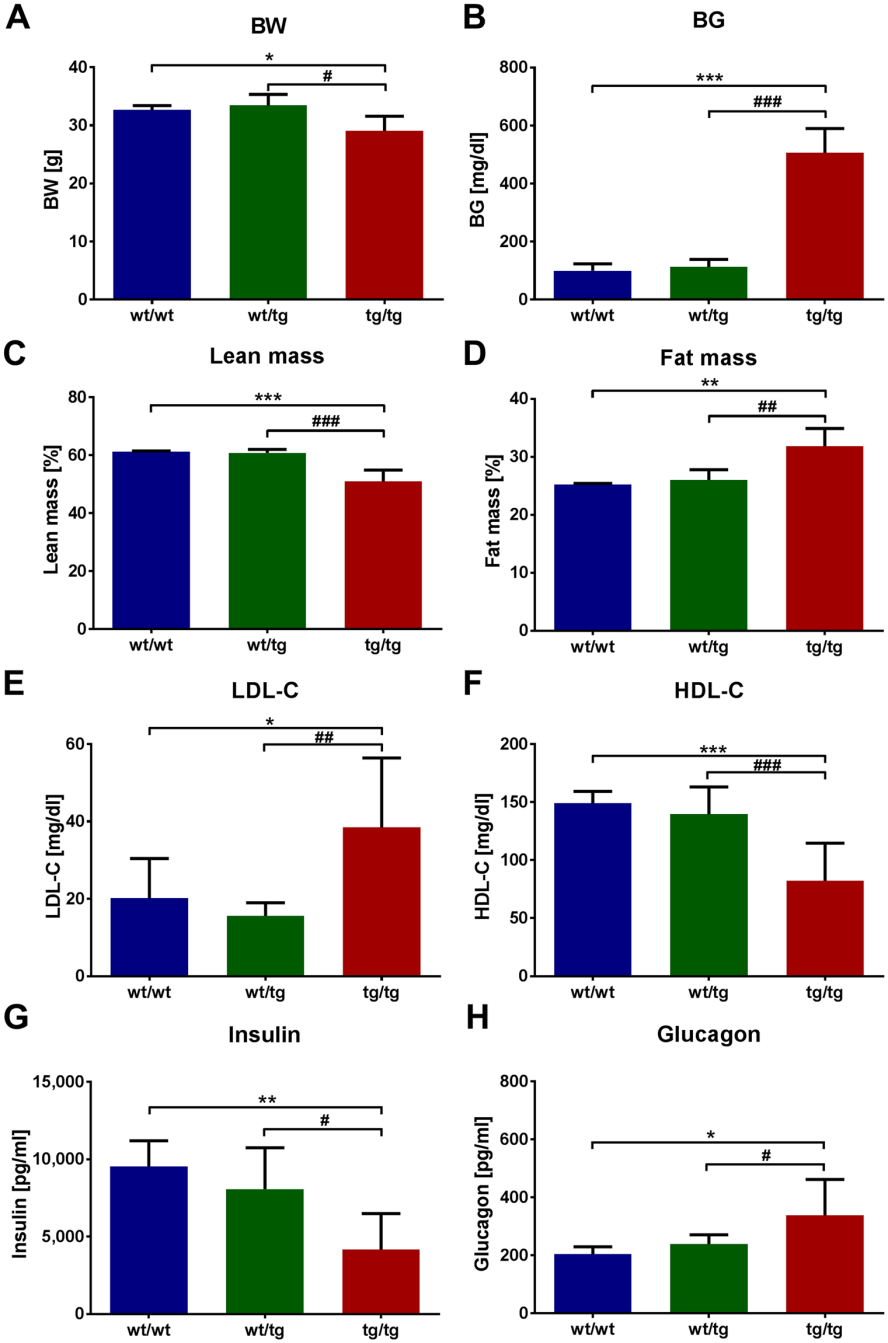
Body weight and plasma parameters of hIAPP mice. (**A**) Body weight and (**B**) fasted blood glucose levels of hIAPP mice. (**C**) Lean and (**D**) fat mass of hIAPP mice depicted in %. Fasted plasma (**E**) LDL-cholesterol (LDL-C) and (**F**) HDL-cholesterol (HDL-C) levels of hIAPP mice. Plasma (**G**) insulin and (**H**) glucagon levels were measured in random fed state. wt/tg and tg/tg denote hemizygous or homozygous transgenic hIAPP mice, respectively. Columns represent averages ± standard deviations; n = 4–11. ^#^Denotes significant differences between wt/tg and tg/tg mice; ^#^p < 0.05, ^##^p < 0.01, ^###^p < 0.001; *Denotes significant differences between wt/wt and tg/tg mice; *p < 0.05, **p < 0.01, ***p < 0.001.

Former studies reported that transgenic mice overexpressing the human form of islet amyloid polypeptide (hIAPP) are prone to develop diabetes^23^. First we characterized the transgenic hIAPP mice, which were shown to specifically overexpress hIAPP in the pancreas^24^. tg/tg mice demonstrated high blood glucose levels and lower body weight compared to wt/wt and wt/tg animals (Fig. 1A,B). Furthermore, tg/tg mice showed reduced lean and elevated fat mass (Fig. 1C,D), which was associated with increased plasma LDL-cholesterol and decreased HDL-cholesterol levels (Fig. 1E,F). Plasma levels of alanine transaminase (ALT), aspartate-aminotransferase (AST), urea and lactate dehydrogenase (LDH) were also elevated in tg/tg animals (Suppl. Figure 1A–D) suggesting liver and kidney damage. Hematoxylin and eosin staining of tg/tg animals demonstrated glomerular mesangial expansion and multifocal protein casts in the kidney (Suppl. Figure 1E) confirming kidney damage. Furthermore, plasma insulin levels were reduced, whereas glucagon levels were elevated in tg/tg animals (Fig. 1G,H), in parallel with the diabetic phenotype.

The lower insulin levels of tg/tg mice suggest pancreatic beta-cell dysfunction, therefore the pancreas was analyzed. Hematoxylin and eosin staining of the pancreas showed less islets and disrupted islet structure in tg/tg animals (Suppl. Figure 1F, right panel). Most islets of wt/tg animals showed normal morphology, however some islets demonstrated cells with reduced cytoplasmic content (Suppl. Figure 1F, middle panel). These results suggest, that the low overexpression of hIAPP in wt/tg mice leads to mild islet morphology changes, while high overexpression of hIAPP in tg/tg mice causes a marked destruction of pancreatic islets.

### hIAPP transgenic mice show amyloid fibrils in the pancreas

**Figure 2:**
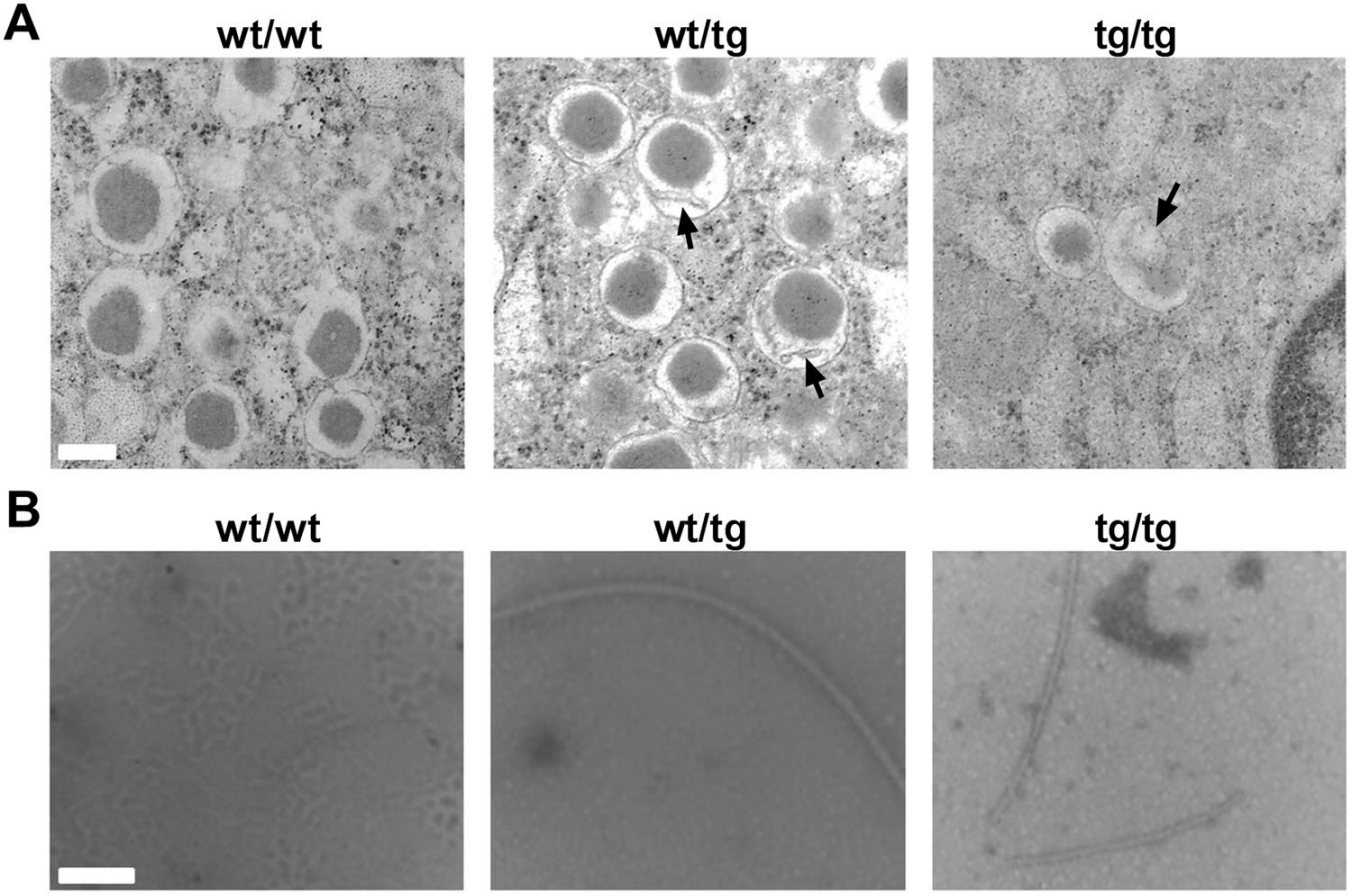
Pancreatic beta-cell morphology and amyloid fibrils isolated from islets of hIAPP mice. (**A**) Pancreatic beta-cells of hIAPP mice were studied using transmission electron microscope. White bar depicts 200 nm, whereas black arrows represent insulin vesicles with aggregates. (**B**) Amyloid fibrils were isolated from pancreatic islets of hIAPP mice and fibrils were visualized using transmission electron microscope (white bar depicts 100 nm). wt/tg and tg/tg denote hemizygous or homozygous transgenic hIAPP mice, respectively. Representative areas are shown, n = 4–6 for A, n = 2–4 for B.

To further analyze the pancreas of the hIAPP mice, transmission electron microscopy was applied, which revealed less and destroyed insulin secretory vesicles with aggregate accumulation in tg/tg mice (Fig. 2A, right panel). This morphology was associated with swollen mitochondria and destroyed cristae structures (data not shown). In wt/tg mice we observed normal beta cell numbers but disturbed insulin secretory vesicle formation with aggregate accumulation (Fig. 2A, middle panel). To identify the structure of these aggregates, amyloid fibrils were isolated from the islets of hIAPP mice. Both transgenic mice (wt/tg and tg/tg) showed long amyloid fibrils in the pancreatic islets (Fig. 2B, middle and right panels). These results suggest that amyloid fibrils are present in both healthy (hemizygous mice) and diabetic state (homozygous mice).

### EGCG interacts with hIAPP *in vitro*

**Figure 3:**
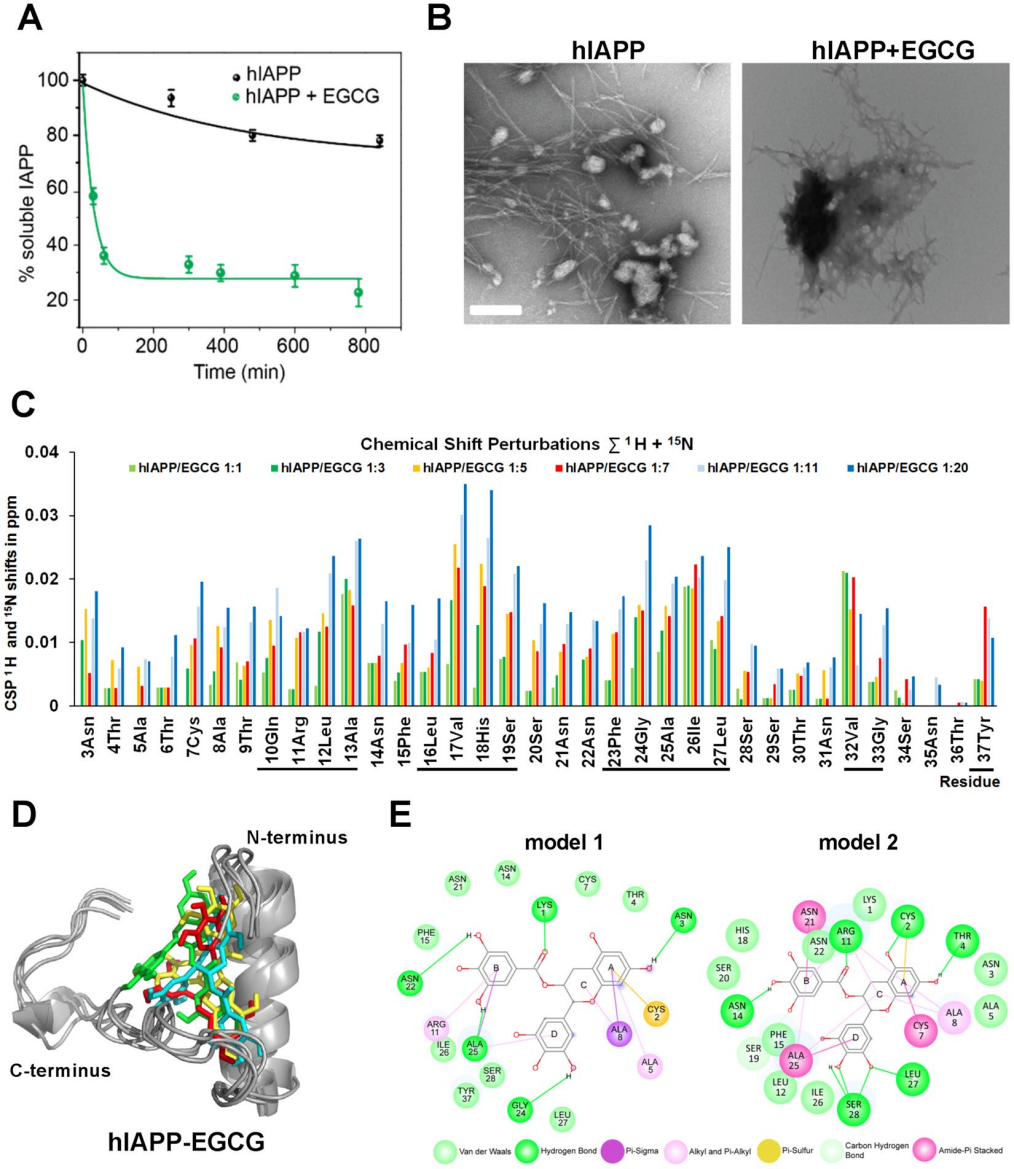
The interaction of EGCG and hIAPP. hIAPP fibril formation was studied for 12–13 hours in the absence or presence of EGCG. (**A**) Aggregation kinetics of hIAPP in the absence (black line) or presence (green line) of EGCG using 1D-NMR. Relative resonance intensities were plotted as a function of time. (**B**) hIAPP fibril formation was studied using transmission electron microscope in the absence (left panel) and presence (right panel) of EGCG. The white bar represents 200 nm. (**A,B**) The molar ratio of hIAPP:EGCG was 1:20. (**C**) Chemical shift perturbations extracted from 2D-NMR experiments. The numbers denote the molar ratio of hIAPP:EGCG. Black bars under the diagram represent the regions of hIAPP, which show the largest changes in chemical shifts upon binding of EGCG. (**D**) Best ranking binding model of hIAPP monomer-EGCG proposed by molecular docking. Various colors represent various binding mode of EGCG obtained by docking. (**E**) hIAPP-EGCG interactions from the HADDOCK docking simulations. The interactions are shown as lines between receptor residues and ligand atoms, the solvent accessible surface of an interacting residue is represented by a halo around the residue. The diameter of the circle is proportional to the solvent accessible surface.

The interaction of hIAPP and EGCG was first analyzed by solution-state NMR. We studied a series of buffers with different composition and pH. We have employed MES buffer (pH 6.5) for all NMR experiments, as hIAPP is not very stable at pH 7.3 using the standard phosphate buffer (Suppl. Figure 2A). After addition of EGCG, the NMR intensities decay over time (Suppl. Figure 2B), which is due to the formation of high molecular weight hIAPP-EGCG complexes followed by aggregation. Also hIAPP alone precipitates out of solution over time, however, the aggregation is much faster in the presence of EGCG (Fig. 3A).

To further investigate the morphological changes in hIAPP induced by addition of EGCG, the hIAPP-EGCG complex was analyzed using transmission electron microscopy. EGCG indeed affects the aggregation process of hIAPP by changing the pathological fibril morphology (Fig. 3B, left panel) to amorphous aggregates (Fig. 3B, right panel), which was also obvious under different pH and buffer conditions (data not shown). EGCG-induced amorphous aggregates have been already shown to not catalyze fibril formation and were postulated to be non-toxic^1^.

To determine which amino acids of hIAPP are responsible for EGCG binding, 2-dimensional NMR experiments have been recorded. After assigning the free hIAPP at pH 6.5, a quantitative analysis was performed, in which EGCG induced chemical shift perturbations (CSPs) of hIAPP were quantified (Suppl. Figure 2C). Figure 3C shows the EGCG-induced chemical shift change. The region of hIAPP, which experience a change in its chemical environment upon addition of EGCG involves residues 10–13, 16–19, 23–27, 32–33 and 37 (Suppl. Figure 2C and Fig. 3C). In principle, CSPs can originate from interactions of hIAPP with EGCG or from hIAPP-hIAPP interactions in the oligomerization process.

To visualize the EGCG-hIAPP complex, we performed molecular docking using HADDOCK. hIAPP is a highly dynamic peptide. The α-helical secondary structure propensity in its N-terminal part is on the order of 40–50%. The docking model must be therefore considered to be transient. As a starting structure, the recently determined hIAPP structural model (PDB accession code 5MGQ^25^) has been employed. The highest scoring models from the top four clusters of docking solutions were analyzed and shown as Fig. 3D. Instead of obtaining only one rigid EGCG-hIAPP complex, we assume that EGCG can interact in many different ways with hIAPP. To indicate this heterogeneity, we have represented an ensemble of docking structural models. All models have in common that EGCG sits in between the coil and the helix in the hIAPP structure (Suppl. Figure 2D). The interaction between hIAPP and EGCG are principally hydrogen bonds, Pi-alkyl, Van-der Waals forces and carbon-hydrogen bonds, which are shown by two interaction models (Fig. 3E). The A ring of EGCG could bind to hIAPP due to hydrogen bonds with Cys2, Asn3, Thr4; due to alkyl and Pi-alkyl with Ala5 and to the Ala8; by Pi-sigma with Ala8; due to amide Pi-stacked with Cys7; by Van der Waals with Asn3, Ala5 and Thr4 and with carbon hydrogen bond with Ala8. The B ring binds to Lys1, Arg11, Asn14, Asn22, and Ala25 via hydrogen bonds; due to alkyl and Pi-alkyl with Arg11; by amide Pi-stacked with Asn21 and Ala25; the Asn14, Phe15, His18, Ser20, Asn21, Asn22 and Ile26 due to Van der Waals. The C ring is in contact with Ala8 due to Pi-sigma binding; due to alkyl and Pi-alkyl with Ala8, Arg11 and Ala25. The D ring is bound via hydrogen bonds with Gly24, Leu27, Ser28; the Ala25 interact by amide Pi-stacked binding; with carbon hydrogen bond with Ser19; and due to Van der Waals with Leu12, Phe15, Ile26, Leu27, Ser28 and Tyr37. We validated the docking results by comparing the structural model to the experimental NMR chemical shift differences for hIAPP in the presence and absence of EGCG. Most of the interacting residues in the highly hydrophobic region of hIAPP show chemical shift perturbations by NMR (Fig. 3C).

### EGCG inhibits amyloid formation of hIAPP *in vitro*

**Figure 4:**
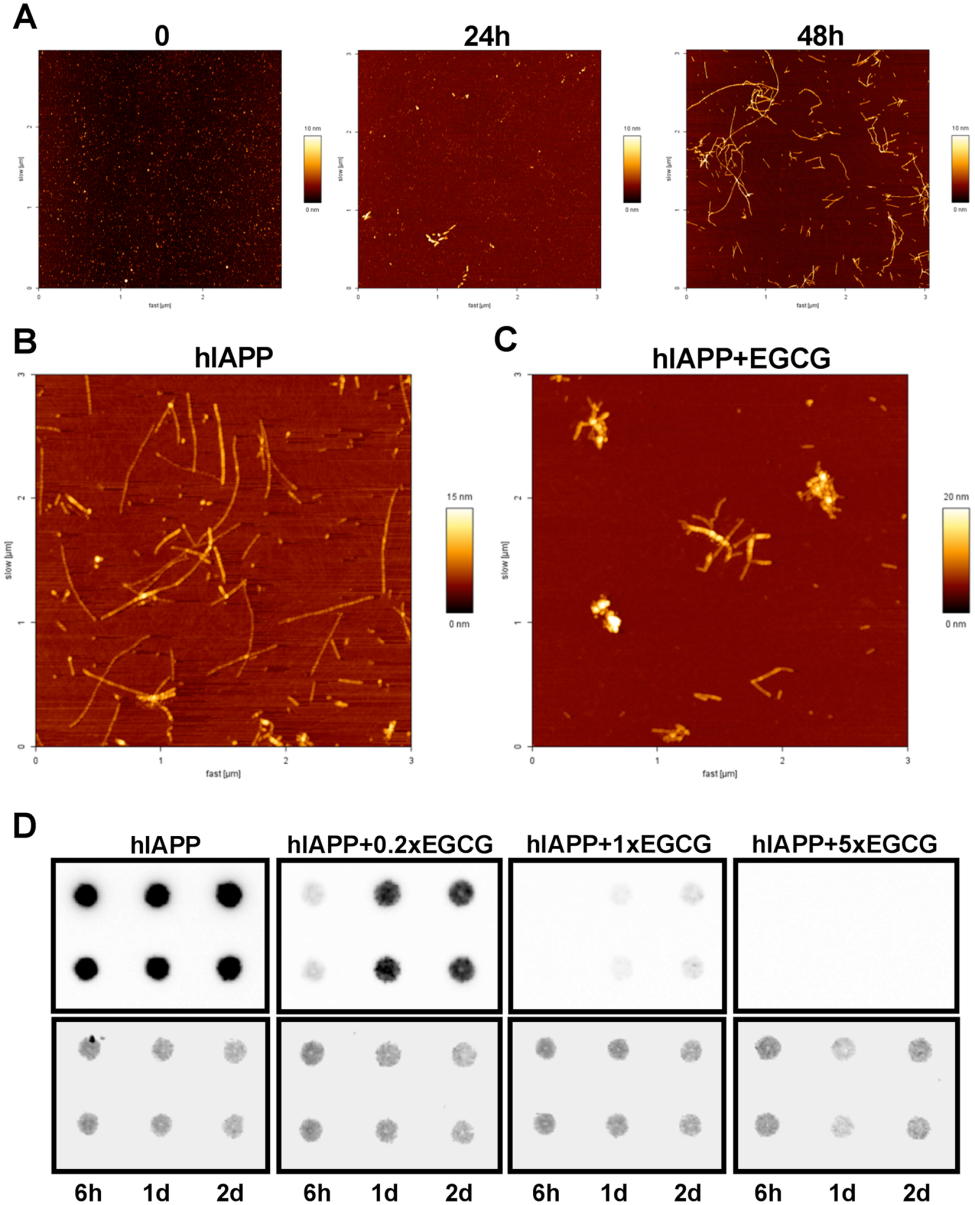
The effect of EGCG on hIAPP amyloid formation *in vitro*. (**A**) Synthetic hIAPP monomers (left panel) forms amyloid oligomers in 24 hours (middle panel) or amyloid fibrils in 48 hours (right panel) analyzed by atomic force microscope (AFM). (**B,C**) hIAPP fibril formation was studied by AFM for 48 hours in the absence (**B**) or presence (**C**) of EGCG. The molar ratio of hIAPP:EGCG was 1:1. Representative areas are shown. Smaller magnifications of the images are shown as Suppl. Figure 3A,B. (**D**) Upper panels: hIAPP fibril formation was studied for the depicted time (h: hours, d: days) in the absence or presence of EGCG using duplicate samples detected by dot blots. The molar ratio of hIAPP:EGCG is depicted above the panels. Lower panels: amidoblack staining was applied for verifying equal protein loading. For one condition two samples are shown from four replicate samples shown as Suppl. Figure 5C.

To study the mechanism of amyloid formation *in vitro*, synthetic hIAPP was generated, which forms oligomers and fibrils in 24 and 48 hours, respectively (Fig. 4A). Since EGCG showed a good inhibitory capacity against amyloid fibrils^9,10^ we applied EGCG to block amyloid formation of hIAPP. In the absence of EGCG, hIAPP formed long amyloid fibrils in 48 hours (Fig. 4B and Suppl. Figure 3A), however in the presence of EGCG, hIAPP formed more amorphous aggregates (Fig. 4C, Suppl. Figure 3B). To differentiate between the early (oligomers) and late (amyloid fibrils) stages of amyloidogenesis, a specific antibody (91D7E8) was generated against amyloid fibrils of hIAPP, which only showed a weak signal for monomers and oligomers, but gave a strong signal for amyloid fibrils (Suppl. Figures 3C and 5A). In order to test the specificity of 91D7E8 antibody, filter retardation assay and dot blot experiments were performed, which showed that 91D7E8 antibody specifically recognizes hIAPP fibrils but not other amyloidogenic proteins *in vitro* as well as it gives a prominent signal in the samples of wt/tg but not in the wt/wt negative control animals (Suppl. Figures 3D,E and 5B). EGCG treatment of hIAPP led to a lower signal of the amyloid fibril specific antibody (data not shown) and increasing EGCG concentration efficiently inhibited hIAPP-formed amyloid fibrils (Fig. 4D and Suppl. Figure 5C). These data suggest that EGCG inhibits the amyloid fibril formation of hIAPP and promotes the formation of more amorphous hIAPP aggregates.

### EGCG reduces amyloid fibril intensity of hIAPP in the pancreas of hIAPP transgenic mice

**Figure 5:**
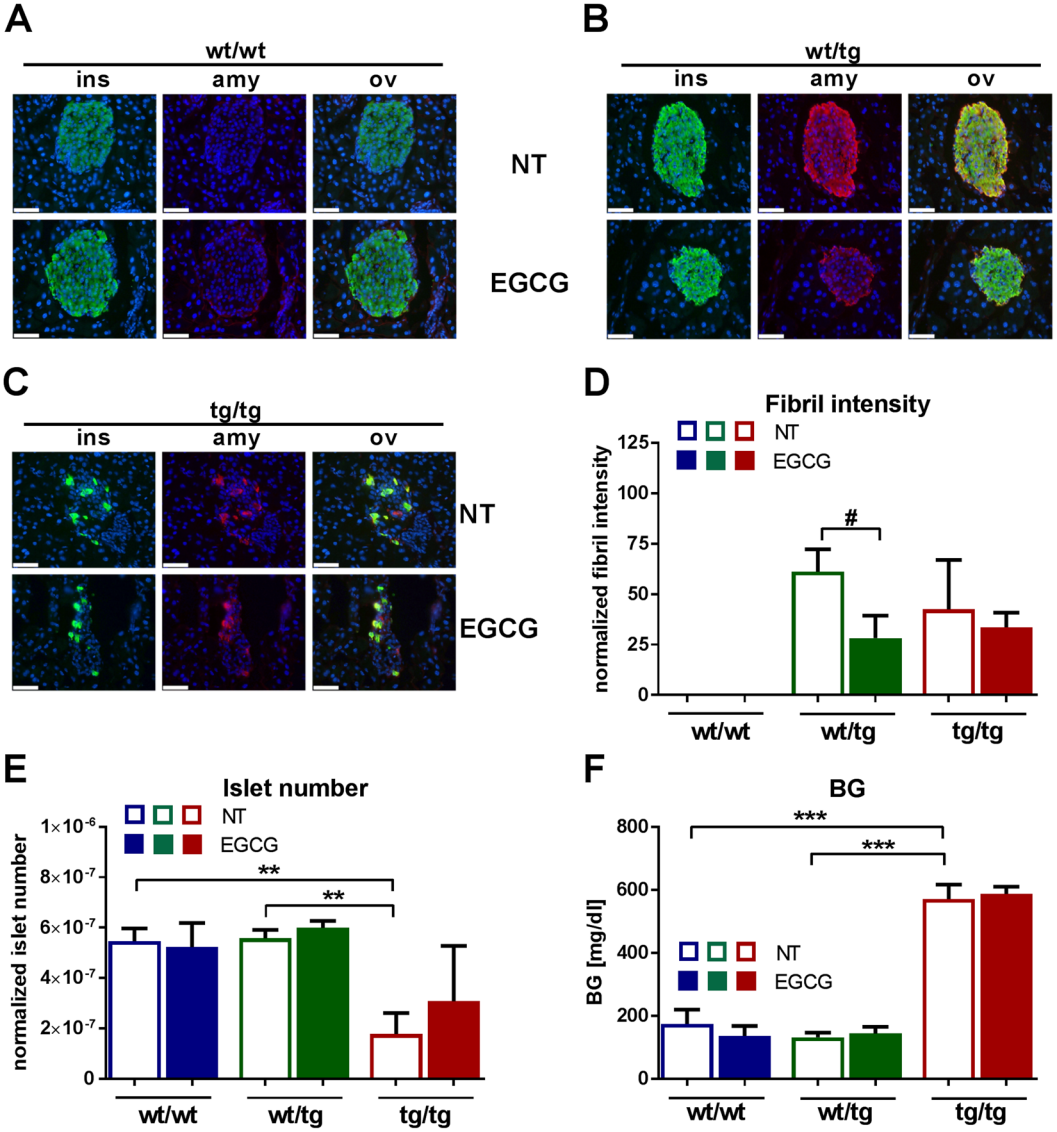
The effect of EGCG on pancreatic islets of hIAPP mice. (**A–C**) Pancreata were stained with anti-insulin (green) and anti-amyloid fibril (91D7E8, red) antibodies and visualized by fluorescence microscopy. Cell nuclei were stained with DAPI (blue), white bars represent 50 µm. ins:insulin, amy:amyloid fibril, ov: overlay, NT:non-treated control group, EGCG:EGCG-treated group. Representative areas are shown. (**D**) The intensity of amyloid fibril staining was calculated using Architect software. (**E**) Pancreatic islet numbers were manually counted and numbers were normalized to total pancreas area. (**F**) Random-fed blood glucose levels. wt/tg and tg/tg denote hemizygous or homozygous transgenic hIAPP mice, respectively. Columns represent averages ± standard deviations; n = 4–7. ^#^Denotes significant differences between wt/tg untreated and wt/tg EGCG-treated mice; ^#^p < 0.05; *Denotes significant differences between wt/wt and tg/tg or wt/tg and tg/tg mice, respectively; **p < 0.01, ***p < 0.001.

In order to inhibit amyloid formation of hIAPP *in vivo*, hIAPP transgenic mice were treated with EGCG for 3 weeks. The hIAPP amyloid fibril specific antibody (91D7E8) showed a pronounced signal in the pancreas of untreated (NT) wt/tg and tg/tg animals, whereas wt/wt control mice showed no staining (Fig. 5A–C, upper panels, red staining). Amyloid fiber staining co-localized with insulin staining (green color) in all conditions (Fig. 5A–C). As it was shown by the hematoxylin and eosin staining (Suppl. Figure 1F), tg/tg mice demonstrated disrupted islet structure, whereas most of the wt/tg animals showed normal islet morphology (Fig. 5B–C, upper panels). EGCG treatment did not change amyloid fibers in tg/tg mice, however it reduced the intensity of amyloid fibers in wt/tg animals (Fig. 5B–D). Insulin and amyloid fiber area remained unchanged upon EGCG application (data not shown). We have found reduced islet numbers and islet area in the pancreas of untreated tg/tg mice, whereas EGCG application showed a tendency to elevate islet numbers and area in these animals (Fig. 5E and Suppl. Figure 4A). Blood glucose levels, body weight and pancreatic insulin intensity were not altered upon EGCG application (Fig. 5F and Suppl. Figure 4B–C). Since EGCG reduced amyloid fiber intensity in wt/tg mice our data indicate that EGCG has a beneficial effect on pancreatic amyloid fibrils *in vivo*.

## Discussion

Although wt/tg hemizygous mice showed normal plasma glucose, insulin, lipid, ALT and AST levels they displayed amyloid aggregates in the insulin secretory vesicles. Biochemical studies revealed that these aggregates are rather long amyloid fibrils. On the other hand homozygous tg/tg mice displayed diabetes, dyslipidemia, decreased insulin, elevated ALT, AST levels and destroyed islet structure. The aggregates in the insulin secretory vesicles of tg/tg mice showed long amyloid fibrils. The 91D7E8 antibody specific for amyloid fibrils verified the presence of fibrils in the pancreatic beta-cells in either wt/tg and tg/tg mice. Interestingly the staining of amyloid fibril showed co-localization with insulin positive cells. Since former detection methods used for detecting amyloid fibrils like Congo red or Thioflavin staining usually identify cells, which are negative for insulin, the 91D7E8 antibody we applied probably recognizes earlier fibrillar structure. These “earlier” amyloid fibrils in tg/tg mice could further develop to higher molecular weight structures, which then could induce membrane rupture and beta-cell death as proposed earlier^4^. At the beginning of hIAPP-induced amyloid plaque research, the late form of amyloidogenesis, in particular amyloid fibrils, were thought to induce toxic intracellular effects in the beta-cells^26,27^. On the other hand later reports revealed that the intermediate form of amyloidogenesis, oligomers, are responsible for cell death found in cell culture and mouse studies^3,28–30^. Therefore, it is conceivable that in the hIAPP mice, amyloid intermediates like oligomers could also participate in the severe beta-cells destruction in tg/tg mice evoking diabetes. It is very challenging to study oligomerization *in vitro*, since the process is very fast and remains almost invisible for biophysical techniques. Since both oligomers and fibrils were previously shown to have toxic effects further studies are needed to determine the exact nature of the toxic amyloid species.

Our data reveal that EGCG interacts with hIAPP and our new binding models show that EGCG can potentially utilize multiple binding modes to bind to the hIAPP monomeric structure. These results are in accordance with earlier studies, suggesting that hIAPP-EGCG binding is a result of multiple mechanisms with different effectiveness depending on the contact. EGCG occupies the binding site formed between the coil and the helix of hIAPP and interacts with most of the protein residues that get perturbed in the NMR spectrum. Combining of biophysical and computational methods, we could identify that the principal residues involved in the interaction between hIAPP and EGCG are R11, L12, S19, A25, I26, L27 and Y37. Former studies using *in silico* approaches identified similar regions for hIAPP-EGCG and hIAPP-hIAPP interactions during aggregation^31,32^. The interaction is mediated by Pi-Pi stacking, Van der Waals, alkyl, Pi-alkyl, conventional hydrogen bonds and carbon hydrogen bond interactions. Initially it was proposed that the inhibition mechanism of EGCG and hIAPP is caused by the interaction with aromatic Pi-Pi stacking bonds^33^, however later studies showed that the trihydroxyl phenyl rings are also important for hIAPP inhibition^13^. Since the inhibition is still active in hIAPP mutants, in which the aromatic residues are changed to leucine, these Pi-Pi interactions are not the only way of binding. This is in agreement with our results, since we found that the EGCG interaction profiles are not only based on Pi-Pi stacking, but also on Van der Waals, alkyl, Pi-alkyl, conventional hydrogen bonds and carbon hydrogen bonds interactions between residue and ligand.

Our data also indicate that the observed chemical shift perturbations in the NMR spectra could be a result of dimerization or polymerization of hIAPP-EGCG molecules. Since dimerization of hIAPP is proposed to be the first step of hIAPP aggregation^31,34,35^ our results indicate that EGCG inhibits hIAPP fibril formation in an early step of amyloidogenic process. Fibrils and toxic oligomers contain a characteristic β-sheet conformation^36–38^, which is lost in newly formed amorphous aggregates^12,13^. Due to the binding of EGCG, the conformation of hIAPP changes: the intra- and intermolecular interactions of hIAPP responsible for β-sheet formation are decreased. A similar mechanism was already proposed for amyloid-β, α-synuclein and immunoglobulin light chain, in which amorphous aggregates are formed in the presence of EGCG as non-toxic species showing a reduced degree of structural homogeneity^9,10,39,40^. In the hIAPP structural analysis^25^, no intermolecular NOE (nuclear Overhauser effect) contacts have been observed, suggesting that the major conformer in our study is monomeric. We cannot rule out that EGCG also interacts with higher molecular weight oligomeric states of hIAPP, as recently reported by Radford and coworkers^41^. However, these oligomers are presumably very low populated and cannot be observed by NMR spectroscopy. In summary, our data indicate that the interaction between EGCG and hIAPP stabilizes the so called amorphous “non-toxic aggregates” and in turn inhibits the formation of toxic oligomers and fibrils.

Application of EGCG decreased the amyloid fibril staining intensity in the pancreas of wt/tg hIAPP mice, suggesting a direct effect of this polyphenol on beta-cells. EGCG was shown to protect insulinoma cells and human islets from cytokine induced apoptosis^42,43^. EGCG also elevated the anti-inflammatory cytokine IL-10 level and decreased blood glucose levels in NOD mice, a mouse model for T1D^43^. Furthermore, glucose stimulated insulin secretion, glucose tolerance and hyperglycemia were improved in EGCG treated db/db mice, which is a model for T2D^44^. These studies suggest that EGCG may have a direct regulatory role on the pancreatic beta-cells. On the other hand, EGCG did not significantly change amyloid fiber intensity in tg/tg mice indicating that EGCG application was ineffective on amyloid fibers in these diabetic mice. Therefore, it is possible that a higher EGCG dose could be beneficial for the treatment of diabetic animals. Furthermore, we observed a tendency to an elevated number of islets and larger islet area in tg/tg mice upon EGCG application, which is in accordance with the study of Ortsater *et al*.^44^.

In summary our data revealed that EGCG has a potential benefit reducing amyloid fibril formation not only *in vitro* but also *in vivo* in wt/tg mice but however it was not efficient to prevent hIAPP aggregation and consequently the diabetic phenotype in tg/tg mice. The hIAPP-EGCG complex structure yields new insights for the design and development of molecules targeting amyloid formation, which are currently under intensive investigation^1^.

## Methods

### Materials

Chemicals were purchased from Sigma-Aldrich (Germany), Roche (Germany) and New England Biolabs (Germany).

### Animal studies

Human islet amyloid polypeptide protein (hIAPP) transgenic mice, which overexpress the hIAPP protein under the control of rat insulin II promoter, were purchased from Jackson Laboratories (USA)^24^. To distinguish between tg/tg and wt/tg mice genomic DNA was isolated and real time PCR was used with the following primers (12852-5′ AGC TGC AAG TAT TTC TCA TTG TG and 12853-3′ TCC GCT TTT CCA CCT GAT G as well as 12855-5′ CAG CGC CTG GCA AAT TTT and 12856-3′ GCA TTC CTC TTG CCA TAT GTA TTG) as published previously^45^. Since female hIAPP TG mice were reported to be protected from diabetes, we only analyzed male mice. Most of the male tg/tg mice develop diabetes. However a rare minority remains normoglycemic, which were excluded in our study. Twenty weeks old male mice were treated with EGCG (Sigma-Aldrich) in the drinking water for 3 weeks. The applied EGCG concentration was 0.4 mg/ml (which approximately corresponds to a dose of 60 mg/kg), untreated mice got normal tap water. Mice were analyzed at the age of 23 weeks, except for pancreatic islet isolation. Since in the long-term diabetic state (at 23 weeks of age), pancreatic islet structure is completely destroyed, islet isolation was not possible in this age (data not shown). Therefore pancreatic islets isolation was performed at 18 weeks of age as described previously^25^. Food and water were available *ad libitum* and mice were killed by isoflurane overdose. All animals received human care and mouse studies were approved by the Institutional Animal Welfare Officer (Helmholtz-Zentrum München) and local government authorities and performed according to GV-SOLAS (Society for Laboratory Animal Science) in accordance with the German Animal Welfare Act.

Plasma alanine transaminase (ALT), aspartate transaminase (AST), urea, lactate dehydrogenase (LDH), low-density lipoprotein cholesterol (LDL-C), high-density lipoprotein cholesterol (HDL-C) levels were quantified using an AU480 clinical chemistry analyzer (Beckman Coulter, Germany)^45^. Blood glucose levels were measured in tail blood samples using a point of care glucometer (Contour, Germany) and plasma insulin and glucagon levels were determined using Bio-Plex Pro mouse diabetes immunoassay (Bio-Rad, Germany). Body composition was studied by quantitative nuclear magnetic resonance (MiniSpec LF60; Bruker Optics, Ettlingen, Germany) as described previously^45,46^.

### Transmission electron microscopy

Pancreata were fixed in 2.5% glutaraldehyde in 0.1 M sodium cacodylate buffer and pancreata as well as amyloid fibers isolated from mouse islets were analyzed as described previously^47^.

### Histochemistry

Liver and kidney tissues were fixed in 4% paraformaldehyde and paraffin sections were stained with hematoxylin and eosin as described previously^48^.

### Nuclear magnetic resonance studies

Human IAPP was produced for solution-state and solid-state nuclear magnetic resonance (NMR) studies and measured as described previously^25,49^. When different buffer or pH compositions were necessary, the powder of hIAPP was solved in 50 µl 30 mM sodium acetate buffer, pH 5.3, followed by the dilution with 10 mM sodium phosphate, pH 7.4 or 5.5; or 20 mM MES (2-(N-morpholino)ethanesulfonic acid), pH 6.5. The final concentration of hIAPP was 100 µM. The fresh EGCG stock solution was solved in pure water and kept at 4 °C. Measurements were done using Avance 500 and 900 MHz spectrometers (Bruker, Germany). Chemical shift assignments have been taken from the peak list of the free monomer, BMRB entry 34069 and to the spectrum recorded at different conditions and transferred by a titration. All data were analyzed using ccpNMR analysis^50^.

### Docking of EGCG to hIAPP

To determine the possible modes of interaction of EGCG with hIAPP, we used High Ambiguity Driven protein-protein DOCKing (HADDOCK). As a starting structure for hIAPP, the recently determined structural model (PDB ID: 5MGQ^25^) was employed. As the differences of the hIAPP chemical shifts in the presence and absence of EGCG are rather small, we performed an unrestrained docking simulation, consisting of randomization of orientations and rigid body energy minimization, which yielded hundreds of solutions. The resulting structures were subjected to semi-rigid simulated annealing in torsion angle space and final refinement in Cartesian space with explicit solvent resulting in ~181 structures that were grouped into 6 clusters. Highest scoring complexes in the top 4 clusters were used for further analysis using Biovia Software D.S. (Discovery Studio Modeling Environment Release 2017, Dassault Systèmes, San Diego, CA, USA). The docking results are validated in retrospect with the experimental NMR chemical shift perturbations.

### hIAPP preparation for dot blots and atomic force microscopy

Synthetic hIAPP peptide was produced by the laboratory of Dr. Volkmar-Engert (Institute for Medical Immunology, Charité, Berlin, Germany) and was solved in 1,1,1,3,3,3-Hexafluoro-2-propanol (HFIP), 10 min sonicated and lyophilized. To produce hIAPP oligomers and fibrils, HFIP-treated hIAPP peptide was solved in 20 mM Tris buffer (pH 7.4), 1 min sonicated and incubated for one day (oligomers) or two days (fibrils) at 37 °C with 300 rpm shaking.

### Atomic force microscopy

Dry atomic force microscope (AFM) images were recorded using a Nanowizard II/Zeiss Axiovert setup (JPK) as described previously^10^.

### Generation of hIAPP amyloid fiber specific antibody (91D7E8)

To generate a monoclonal antibody against hIAPP amyloid fibrils, synthetic human lyophilized HFIP-treated hIAPP was solved in 20 mM Tris buffer (pH 7.4) to a concentration of 30 µM. The solution was sonicated for 1 min and incubated for 2 days at 37 °C with shaking at 300 rpm. The resulting fibrils were used for antibody generation according to a proprietary immunization protocol of Synaptic Systems Göttingen (see also http://www.sysy.com/mabservice.html): Three 8–10 week-old BALB/c female mice were subcutaneously immunized with hIAPP fibrils over a period of 17 days. Cells from the knee lymph nodes were fused with the mouse myeloma cell line P3X63Ag8.653 (ATCC CRL-1580). The clones used in this study were re-cloned two times by limiting dilution and the immunoglobulin subclass was determined. Antibodies secreted by the hybridomas were screened for their reactivity against the immunogen using ELISA. Positive antibodies were retested by ELISA against hIAPP fibrils and monomer. Hybridomas producing antibodies with preference for hIAPP fibrils were subcloned to monoclonality and further analyzed for histology and western blot applications. One of the antibodies showing high specificity for hIAPP fibrils was the antibody 91D7E8, which was chosen for the further experiments.

### Dot blot assay

To analyze the specificity of anti-fibril (91D7E8) antibody, hIAPP monomer (HFIP-treated hIAPP peptide solved in 20 mM Tris buffer (pH 7.4), 1 min sonicated), oligomer and fibril solutions were spotted onto nitrocellulose membrane and incubated with anti-fibril antibody as published previously^25^. Furthermore, 30 µM hIAPP was incubated with different concentrations of EGCG in 20 mM Tris-HCl (pH 7.4) at 37 °C with 300 rpm shaking. As a control, hIAPP was incubated with the compound solvent (DMSO). After 0 hour, 6 hours, 1, 2 and 3 days of incubation, samples were analyzed with dot blot using anti-fibril (91D7E8) antibody. Per dot 10 µl of sample was analyzed.

### Native filter retardation assay

Frozen pancreatic tissues was homogenized in a 5-fold excess (w/v) of ice cold 50 mM Tris-HCl pH 7.5, 150 mM NaCl, 0.1% SDS, 0.5% Sodium deoxycholate, 1% Triton X-100, 0.25 U/µl Benzonase and complete protease inhibitor cocktail using a Schütt Homgen Plus semi-automatic homogenizer (700 rpm).

Filter retardation assay was performed as published previously^51,52^. Samples were filtered through a cellulose acetate membrane with a pore size of 0.2 µm (GE Healthcare Life Sciences, Germany). After washing the membrane with 1× PBS (13.7 mM NaCl, 0.27 mM KCl, 1 mM Na_2_HPO_4_, 0.2 mM KH_2_PO_4_, pH 7.4), it was blocked for 30 min with 3% skim milk (Sigma-Aldrich, Germany) in 1× PBS containing 0.05% Tween 20 (PBS-T). Then, the membrane was incubated over night with the 91D7E8 antibody (1:1000) diluted in 3% skim milk PBS-T. Subsequently, the membrane was washed and incubated with the secondary peroxidase-conjugated anti-mouse antibody for 1 h. After washing, the immunoreactive protein was detected using ChemiGlow (Biozym, Germany) and FujiFilm LAS-3000 (Fuji, Japan).

### Immunofluorescence staining

Pancreata were fixed in 4% paraformaldehyde and cryosections were stained with anti-fibril (91D7E8), anti-insulin antibodies and cell nuclei with DAPI staining as described previously^48^. Slides were scanned using a NanoZoomer 2.0 HT (Hamamatsu, Japan) fluorescence scanner; pancreatic islets were evaluated using Definiens Developer XD 2 image analysis software (Definiens AG, Germany) as described previously^45^. Briefly, the border of the islets was manually marked and DAPI staining identified cell nuclei with insulin or amyloid fiber positivity as well as insulin and amyloid fiber intensities were calculated by Definiens software. First an average was built for insulin or amyloid values for each animals one by one, and as a second step an average was built for each treatment group (n:4–7). Islet numbers were manually counted and islet numbers as well as islet areas were normalized to pancreatic areas. The intensities of amyloid fibril staining were normalized to the values of wild-type wt/wt mice.

### Statistics

Statistical evaluations were performed using GraphPad Prism. ANOVA with post hoc Holm-Šídák’s multiple comparison tests were used to calculate statistical significance, which was assumed at p < 0.05.

## Electronic supplementary material

Supplementary Figures

## References

[1] Bieschke J (2013). Natural compounds may open new routes to treatment of amyloid diseases. Neurotherapeutics.

[2] Ankarcrona M (2016). Current and future treatment of amyloid diseases. J. Intern. Med..

[3] Zraika S (2010). Toxic oligomers and islet beta cell death: guilty by association or convicted by circumstantial evidence?. Diabetologia.

[4] Westermark P, Andersson A, Westermark GT (2011). Islet amyloid polypeptide, islet amyloid, and diabetes mellitus. Physiol. Rev..

[5] Pithadia A, Brender JR, Fierke CA, Ramamoorthy A (2016). Inhibition of IAPP Aggregation and Toxicity by Natural Products and Derivatives. Journal of diabetes research.

[6] Zraika S (2009). Oxidative stress is induced by islet amyloid formation and time-dependently mediates amyloid-induced beta cell apoptosis. Diabetologia.

[7] Johnson KH, O’Brien TD, Jordan K, Westermark P (1989). Impaired glucose tolerance is associated with increased islet amyloid polypeptide (IAPP) immunoreactivity in pancreatic beta cells. Am. J. Pathol..

[8] Brender JR, Salamekh S, Ramamoorthy A (2012). Membrane disruption and early events in the aggregation of the diabetes related peptide IAPP from a molecular perspective. Acc. Chem. Res..

[9] Ehrnhoefer DE (2008). EGCG redirects amyloidogenic polypeptides into unstructured, off-pathway oligomers. Nat. Struct. Mol. Biol..

[10] Bieschke J (2010). EGCG remodels mature alpha-synuclein and amyloid-beta fibrils and reduces cellular toxicity. Proc. Natl. Acad. Sci. USA.

[11] Huang R (2012). NMR characterization of monomeric and oligomeric conformations of human calcitonin and its interaction with EGCG. J. Mol. Biol..

[12] Meng F, Abedini A, Plesner A, Verchere CB, Raleigh DP (2010). The flavanol (−)-epigallocatechin 3-gallate inhibits amyloid formation by islet amyloid polypeptide, disaggregates amyloid fibrils, and protects cultured cells against IAPP-induced toxicity. Biochemistry.

[13] Cao P, Raleigh DP (2012). Analysis of the inhibition and remodeling of islet amyloid polypeptide amyloid fibers by flavanols. Biochemistry.

[14] Suzuki Y, Brender JR, Hartman K, Ramamoorthy A, Marsh EN (2012). Alternative pathways of human islet amyloid polypeptide aggregation distinguished by (19)f nuclear magnetic resonance-detected kinetics of monomer consumption. Biochemistry.

[15] Popovych N (2012). Site specific interaction of the polyphenol EGCG with the SEVI amyloid precursor peptide PAP(248–286). J. Phys. Chem. B.

[16] Yan J, Zhao Y, Suo S, Liu Y, Zhao B (2012). Green tea catechins ameliorate adipose insulin resistance by improving oxidative stress. Free Radic. Biol. Med..

[17] Chen YK (2011). Effects of green tea polyphenol (-)-epigallocatechin-3-gallate on newly developed high-fat/Western-style diet-induced obesity and metabolic syndrome in mice. J. Agric. Food Chem..

[18] Legeay S, Rodier M, Fillon L, Faure S, Clere N (2015). Epigallocatechin Gallate: A Review of Its Beneficial Properties to Prevent Metabolic Syndrome. Nutrients.

[19] Hosoda K (2003). Antihyperglycemic effect of oolong tea in type 2 diabetes. Diabetes Care.

[20] Fukino Y (2008). Randomized controlled trial for an effect of green tea-extract powder supplementation on glucose abnormalities. Eur. J. Clin. Nutr..

[21] Mackenzie T, Leary L, Brooks WB (2007). The effect of an extract of green and black tea on glucose control in adults with type 2 diabetes mellitus: double-blind randomized study. Metabolism.

[22] Verchere CB (1996). Islet amyloid formation associated with hyperglycemia in transgenic mice with pancreatic beta cell expression of human islet amyloid polypeptide. Proc. Natl. Acad. Sci. USA.

[23] Janson J (1996). Spontaneous diabetes mellitus in transgenic mice expressing human islet amyloid polypeptide. Proc. Natl. Acad. Sci. USA.

[24] Jackson-Laboratory. Mouse Strain Datasheet-008232: FVB/N-Tg(Ins2-IAPP)RHFSoel/J. https://www.jax.org/strain/008232 (2017).

[25] Rodriguez Camargo DC (2017). The redox environment triggers conformational changes and aggregation of hIAPP in Type IIDiabetes. Sci. Rep..

[26] Westermark P (1994). Amyloid and polypeptide hormones: What is their interrelationship?. Amyloid.

[27] Hoppener JW, Ahren B, Lips CJ (2000). Islet amyloid and type 2 diabetes mellitus. N. Engl. J. Med..

[28] Bram Y (2014). Apoptosis induced by islet amyloid polypeptide soluble oligomers is neutralized by diabetes-associated specific antibodies. Sci. Rep..

[29] Abedini, A. *et al*. Time-resolved studies define the nature of toxic IAPP intermediates, providing insight for anti-amyloidosis therapeutics. *eLife* **5**, 10.7554/eLife.12977 (2016). 10.7554/eLife.12977 PMC4940161 27213520

[30] Aitken JF (2017). Rutin suppresses human-amylin/hIAPP misfolding and oligomer formation *in-vitro*, and ameliorates diabetes and its impacts in human-amylin/hIAPP transgenic mice. Biochem. Biophys. Res. Commun..

[31] Mo Y, Lei J, Sun Y, Zhang Q, Wei G (2016). Conformational Ensemble of hIAPP Dimer: Insight into the Molecular Mechanism by which a Green Tea Extract inhibits hIAPP Aggregation. Sci. Rep..

[32] Wang Q, Guo J, Jiao P, Liu H, Yao X (2014). Exploring the influence of EGCG on the beta-sheet-rich oligomers of human islet amyloid polypeptide (hIAPP1-37) and identifying its possible binding sites from molecular dynamics simulation. PLoS One.

[33] Porat Y, Abramowitz A, Gazit E (2006). Inhibition of amyloid fibril formation by polyphenols: structural similarity and aromatic interactions as a common inhibition mechanism. Chem. Biol. Drug Des..

[34] Bram Y (2015). Monitoring and targeting the initial dimerization stage of amyloid self-assembly. Angew. Chem. Int. Ed. Engl..

[35] Wiltzius JJ, Sievers SA, Sawaya MR, Eisenberg D (2009). Atomic structures of IAPP (amylin) fusions suggest a mechanism for fibrillation and the role of insulin in the process. Protein Sci..

[36] Weirich F (2016). Structural Characterization of Fibrils from Recombinant Human Islet Amyloid Polypeptide by Solid-State NMR: The Central FGAILS Segment Is Part of the beta-Sheet Core. PLoS One.

[37] Heise H (2005). Molecular-level secondary structure, polymorphism, and dynamics of full-length alpha-synuclein fibrils studied by solid-state NMR. Proc. Natl. Acad. Sci. USA.

[38] Luca S, Yau WM, Leapman R, Tycko R (2007). Peptide conformation and supramolecular organization in amylin fibrils: constraints from solid-state NMR. Biochemistry.

[39] Lopez del Amo JM (2012). Structural properties of EGCG-induced, nontoxic Alzheimer’s disease Abeta oligomers. J. Mol. Biol..

[40] Hora M (2017). Epigallocatechin-3-gallate preferentially induces aggregation of amyloidogenic immunoglobulin light chains. Sci. Rep..

[41] Young LM, Cao P, Raleigh DP, Ashcroft AE, Radford SE (2014). Ion mobility spectrometry-mass spectrometry defines the oligomeric intermediates in amylin amyloid formation and the mode of action of inhibitors. J. Am. Chem. Soc..

[42] Zhang Z, Ding Y, Dai X, Wang J, Li Y (2011). Epigallocatechin-3-gallate protects pro-inflammatory cytokine induced injuries in insulin-producing cells through the mitochondrial pathway. Eur. J. Pharmacol..

[43] Fu Z, Zhen W, Yuskavage J, Liu D (2011). Epigallocatechin gallate delays the onset of type 1 diabetes in spontaneous non-obese diabetic mice. Br. J. Nutr..

[44] Ortsater H, Grankvist N, Wolfram S, Kuehn N, Sjoholm A (2012). Diet supplementation with green tea extract epigallocatechin gallate prevents progression to glucose intolerance in db/db mice. Nutr Metab (Lond).

[45] Franko A (2016). Bezafibrate Improves Insulin Sensitivity and Metabolic Flexibility in STZ-Induced Diabetic Mice. Diabetes.

[46] Tschop MH (2012). A guide to analysis of mouse energy metabolism. Nat Methods.

[47] Franko A (2013). Efficient Isolation of Pure and Functional Mitochondria from Mouse Tissues Using Automated Tissue Disruption and Enrichment with Anti-TOM22 Magnetic Beads. PLoS One.

[48] Franko A (2017). Bezafibrate ameliorates diabetes via reduced steatosis and improved hepatic insulin sensitivity in diabetic TallyHo mice. Molecular metabolism.

[49] Rodriguez Camargo DC (2015). Cloning, expression and purification of the human Islet Amyloid Polypeptide (hIAPP) from Escherichia coli. Protein Expr. Purif..

[50] Vranken WF (2005). The CCPN data model for NMR spectroscopy: development of a software pipeline. Proteins.

[51] Wanker EE (1999). Membrane filter assay for detection of amyloid-like polyglutamine-containing protein aggregates. Methods Enzymol..

[52] Heiser V (2002). Identification of benzothiazoles as potential polyglutamine aggregation inhibitors of Huntington’s disease by using an automated filter retardation assay. Proc. Natl. Acad. Sci. USA.

