# Supplementary material for: Epigallocatechin gallate (EGCG) reduces the intensity of pancreatic amyloid fibrils in human islet amyloid polypeptide (hIAPP) transgenic mice
Source: Sci Rep. 2018 Jan 18;8:1116. doi: 10.1038/s41598-017-18807-8 (PMC5773570; doi:10.1038/s41598-017-18807-8)
Supplement: Supplementary file 1 — Supplementary Figures [file 41598_2017_18807_MOESM1_ESM.pdf]

Epigallocatechin gallate (EGCG) reduces the intensity of pancreatic amyloid fibrils in human islet amyloid polypeptide (hIAPP) transgenic mice

Andras Franko, Diana C. Rodriguez Camargo, Annett Böddrich, Divita Garg, Andres Rodriguez Camargo, Birgit Rathkolb, Dirk Janik, Michaela Aichler, Annette Feuchtinger, Frauke Neff, Helmut Fuchs, Erich E. Wanker, Bernd Reif, Hans-Ulrich Häring, Andreas Peter, Martin Hrabě de Angelis

Supplementary Information

Supplementary Figure 1 Plasma parameters as well as kidney and pancreas morphology of hIAPP mice

Plasma **A** alanine transaminase (ALT), **B** aspartate aminotransferase (AST), **C** urea and **D** lactate dehydrogenase (LDH) levels were measured using clinical chemistry. **E** Kidney morphology was investigated by hematoxilin and eosin staining, white bar represents 200 µm. Black arrows indicate glomerular mesangial expansion, asterisks denote multifocal protein casts. **F** Pancreatic islet morphology of hIAPP mice was assessed using hematoxilin and eosin staining. White bar depicts 100 µm, whereas white squares represent cells showing reduced cytoplasmic content. Representative areas are shown. wt/tg and tg/tg denote hemizygous or homozygous transgenic hIAPP mice, respectively. Columns represent averages ± standard deviations; n=4-10. #denotes significant differences between wt/tg and tg/tg mice; ###$p<0.001$; *denotes significant differences between wt/wt and tg/tg mice; ***$p<0.001$.

Supplementary Figure 2 The interaction of EGCG and hIAPP using NMR

A The relative intensity of 1D-NMR spectra is proportional to the amount of hIAPP peptide in solution. **B** A representative 1D-NMR spectrum of hIAPP in the absence (black line) or presence (green line) of EGCG in MES buffer, pH 6.5. The molar ratio of hIAPP:EGCG was 1:20. **C** 2-dimensional ^{1}H, ^{15}N spectrum of hIAPP in the absence (black areas) or presence (green areas) of EGCG. The molar ratio of hIAPP:EGCG was 1:20. **D** Hydrophobicity plot of the hIAPP-EGCG complex. Blue denotes hydrophilic, white denotes 0, red denotes hydrophobic residues.

Supplementary Figure 3 The interaction of EGCG and hIAPP using AFM as well as specificity test of 91D7E8 antibody using dot blot and filter retardation assay

hIAPP fibril formation was studied by atomic force microscope for 48 hours in the absence **A** or presence **B** of EGCG. The molar ratio of hIAPP:EGCG was 1:1. White squares are shown

as Figure 4B-C. Representative areas are shown. **C** A new antibody (91D7E8) was generated against hIAPP fibrils and tested on amyloid monomers, oligomers and fibrils using dot blot with the indicated antibody dilutions. Amidoblack staining was applied for verifying equal protein loading. For one condition one sample is shown from one-three replicate samples shown as Suppl. Fig. 5A. **D** Filter retardation assay incubated with pancreatic samples of hIAPP mice and labeled with 91D7E8 antibody. Pancreas homogenates of 4-6 different mice in triplicates were tested. **E** Dot blot analysis (right panel) and its amidoblack stained membrane (left panel) of different monomeric and fibrillar amyloid proteins incubated with 91D7E8 antibody. aβ: amyloid-beta; αsyn: alpha-synuclein, τ40:tau 40 protein; mono: monomer; fib: fibril. For one condition one sample is shown from three-four replicate samples shown as Suppl. Fig. 5B.

Supplementary Figure 4 Pancreatic islet morphology and body weight of hIAPP mice

A Islet area normalized to pancreas area and **B** insulin intensity were calculated using Architect software. **C** Body weight of hIAPP mice. wt/tg and tg/tg denote hemizygous or homozygous transgenic hIAPP mice, respectively. Columns represent averages ± standard deviations; n=4-10. *denotes significant differences between wt/wt and tg/tg mice, *$p<0.05$.

Supplementary Figure 5 The interaction of EGCG and hIAPP using 91D7E8 antibody in dot blots

A 91D7E8 antibody was used on hIAPP amyloid monomers, oligomers and fibrils in dot blot with the indicated antibody dilutions. Amidoblack staining was applied for verifying equal protein loading. Triplicate samples were applied for monomers and fibrils, oligomers were used as single samples. **B** Dot blot analysis (right panel) and its amidoblack stained membrane (left panel) of different monomeric and fibrillar amyloid proteins incubated with 91D7E8 antibody. aβ: amyloid-beta; αsyn: alpha-synuclein, τ40:tau 40 protein; mono: monomer; fib: fibril. Three-four replicate samples are shown. **C** Left panel: hIAPP fibril formation was studied for the depicted time (h: hours, d: days) in the absence or presence of EGCG by dot blots. 0 minute samples were collected on ice after pipetting have been completed for all samples. The molar ratio of hIAPP:EGCG is depicted above/below the panels. Right panel: amidoblack staining was applied for verifying equal protein loading. Four replicate samples are shown.

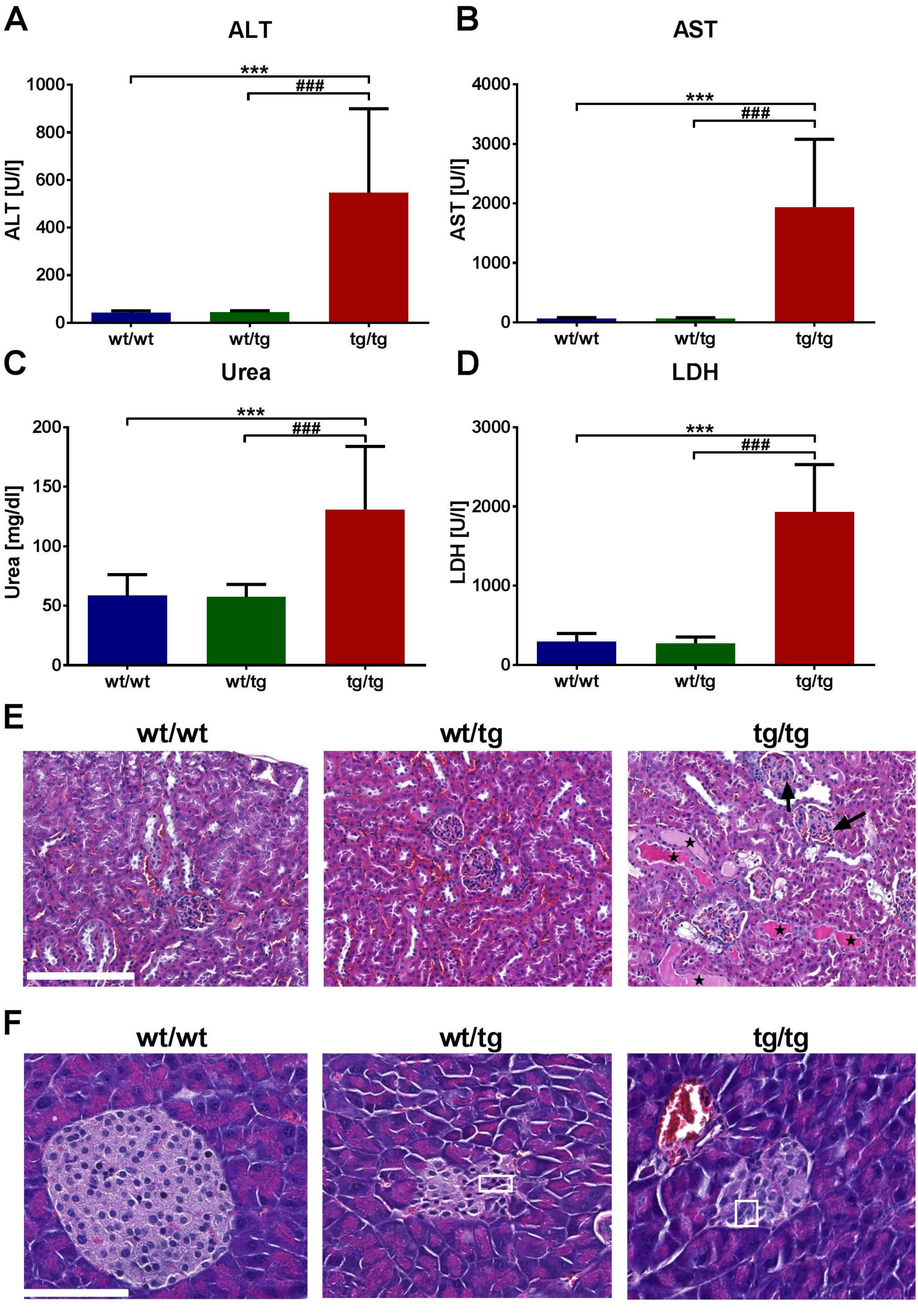

Supplementary Figure 1

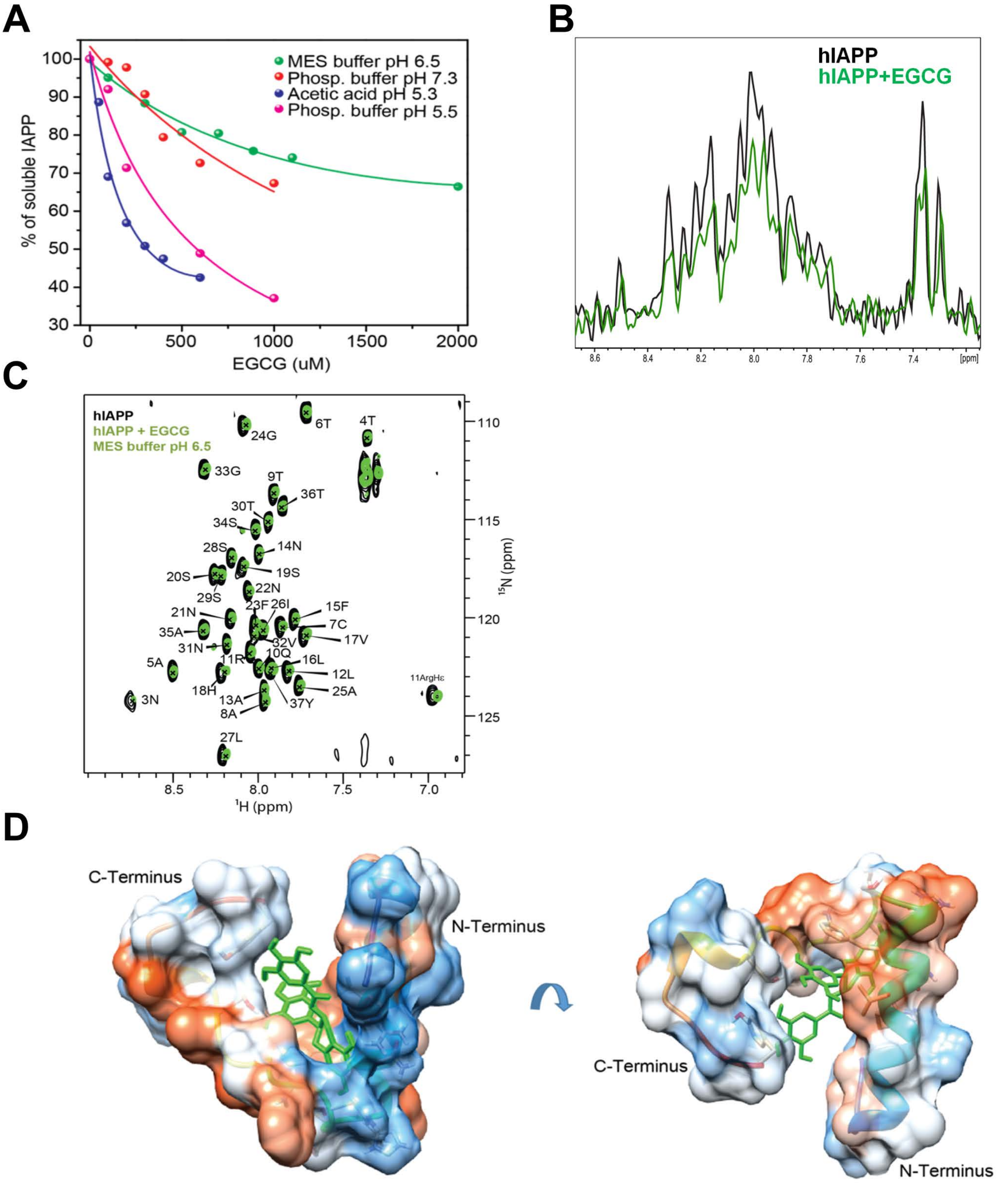

Supplementary Figure 2

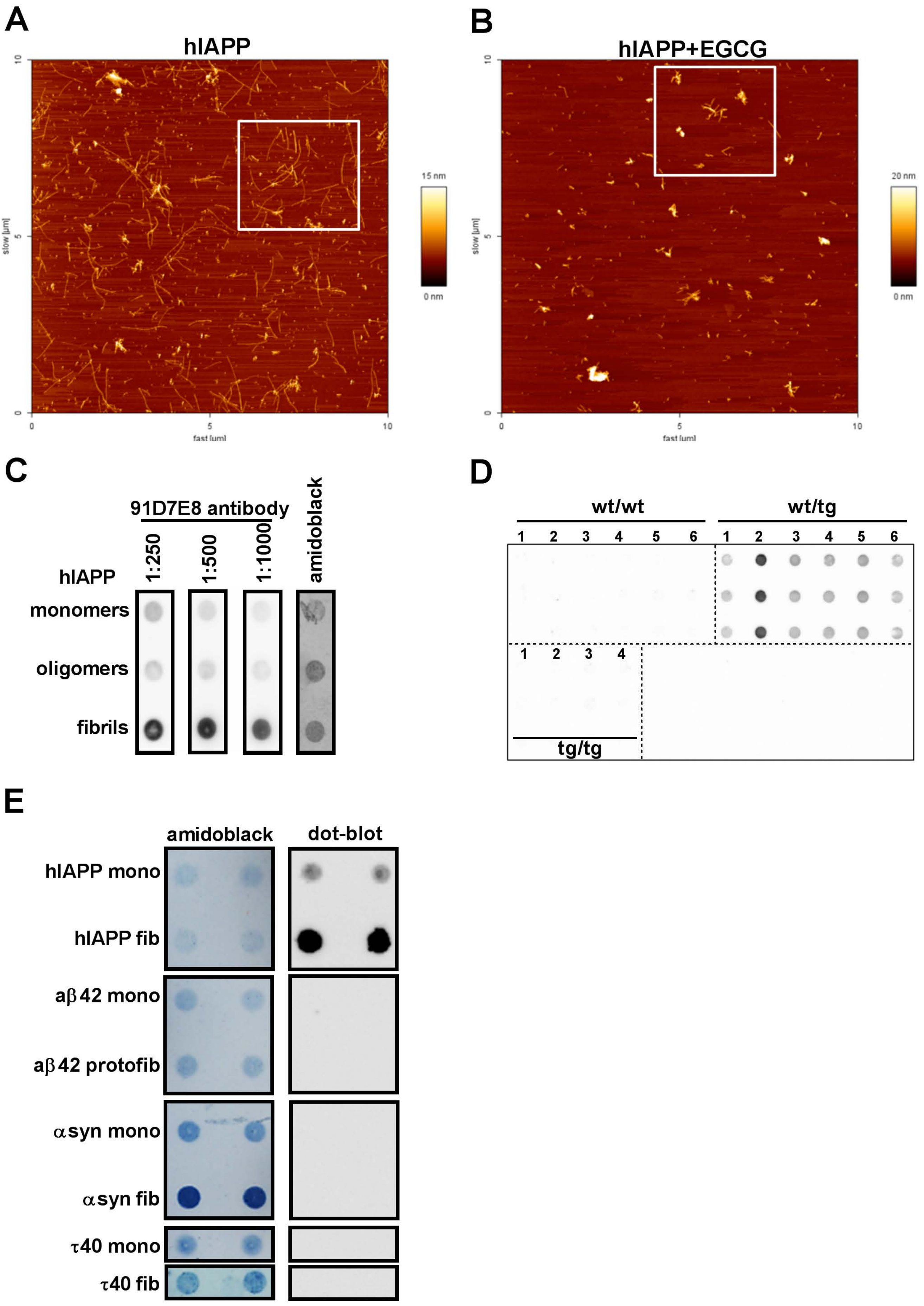

Supplementary Figure 3

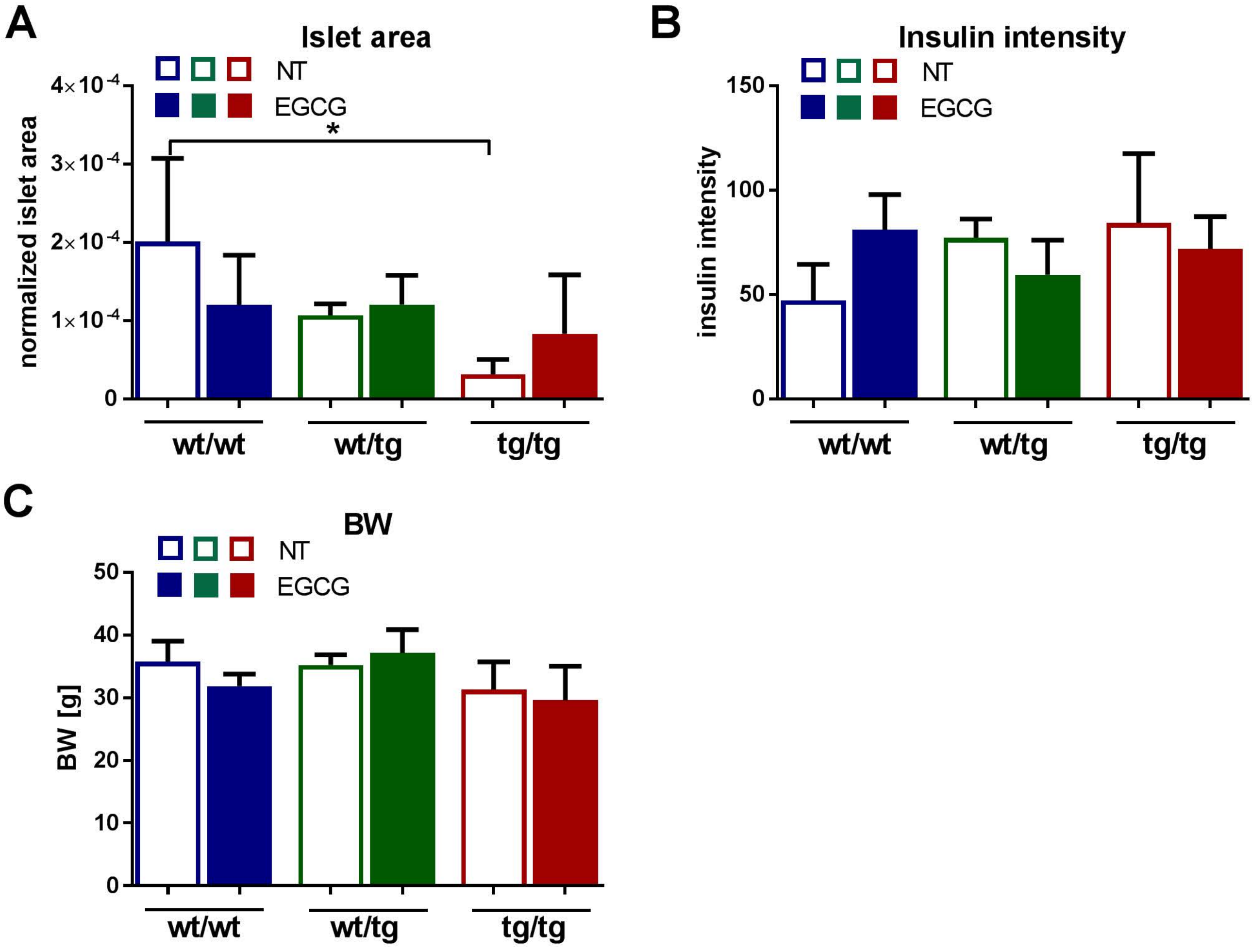

Supplementary Figure 4

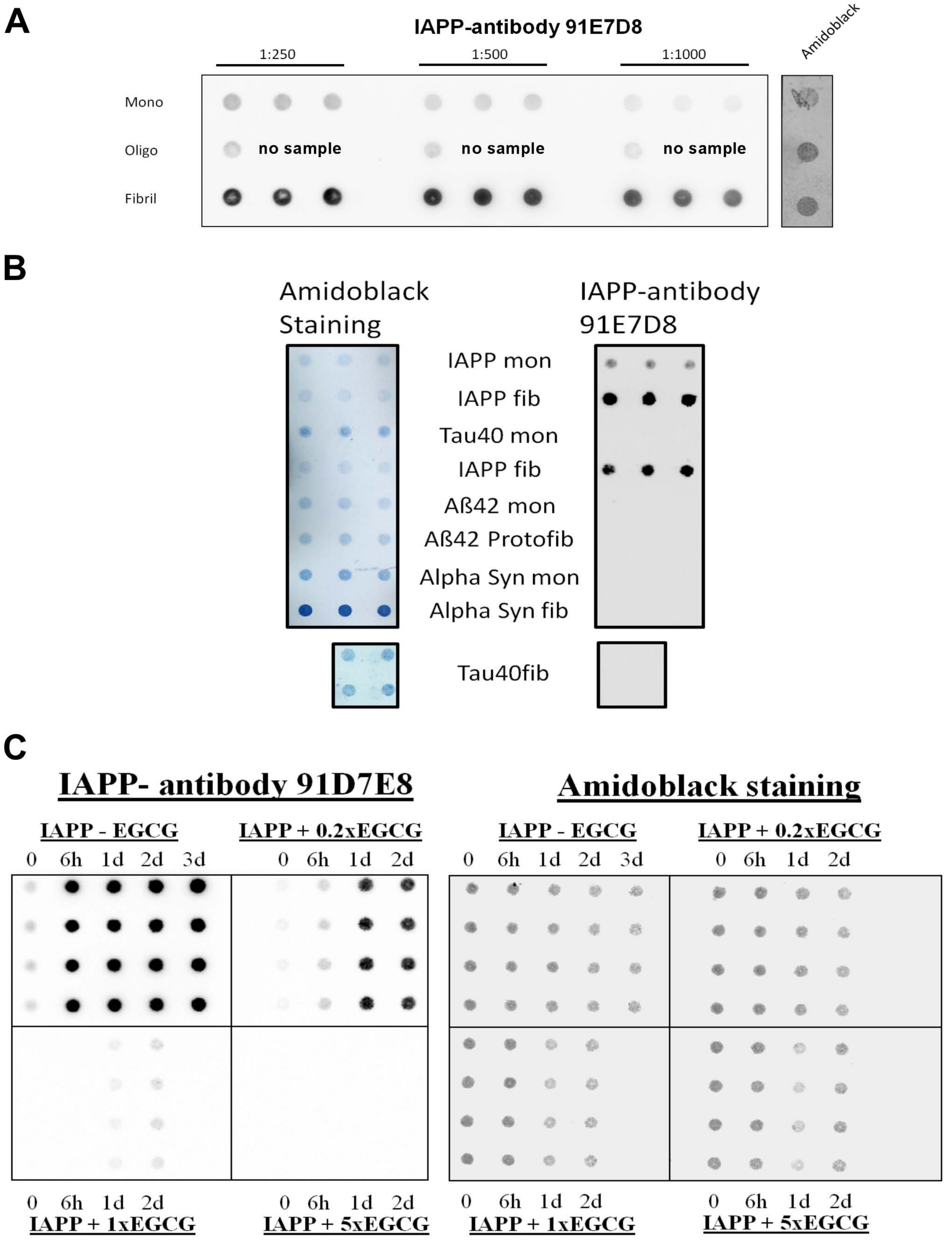

Supplementary Figure 5
